# Impact of community-based support services on antiretroviral treatment programme delivery and outcomes in resource-limited countries: a synthetic review

**DOI:** 10.1186/1472-6963-12-194

**Published:** 2012-07-09

**Authors:** Edwin Wouters, Wim Van Damme, Dingie van Rensburg, Caroline Masquillier, Herman Meulemans

**Affiliations:** 1 Department of Sociology and Research Centre for Longitudinal and Life Course Studies, University of Antwerp, Sint-Jacob Street 2, 2000, Antwerp, Belgium; 2 Centre for Health Systems Research and Development, University of the Free State, Nelson Mandela Avenue, Bloemfontein, South Africa; 3 Unit of Health Policy and Financing, Department of Public Health, Institute of Tropical Medicine, Sint-Rochusstraat 43, 2000, Antwerp, Belgium

**Keywords:** HIV/AIDS, Antiretroviral treatment, Community support, Lay health workers, Resource-limited countries

## Abstract

**Background:**

Task-shifting to lay community health providers is increasingly suggested as a potential strategy to overcome the barriers to sustainable antiretroviral treatment (ART) scale-up in high-HIV-prevalence, resource-limited settings. The dearth of systematic scientific evidence on the contributory role and function of these forms of community mobilisation has rendered a formal evaluation of the published results of existing community support programmes a research priority.

**Methods:**

We reviewed the relevant published work for the period from November 2003 to December 2011 in accordance with the guidelines for a synthetic review. ISI Web of Knowledge, Science Direct, BioMed Central, OVID Medline, PubMed, Social Services Abstracts, and Sociological Abstracts and a number of relevant websites were searched.

**Results:**

The reviewed literature reported an unambiguous positive impact of community support on a wide range of aspects, including access, coverage, adherence, virological and immunological outcomes, patient retention and survival. Looking at the mechanisms through which community support can impact ART programmes, the review indicates that community support initiatives are a promising strategy to address five often cited challenges to ART scale-up, namely (1) the lack of integration of ART services into the general health system; (2) the growing need for comprehensive care, (3) patient empowerment, (4) and defaulter tracing; and (5) the crippling shortage in human resources for health. The literature indicates that by linking HIV/AIDS-care to other primary health care programmes, by providing psychosocial care in addition to the technical-medical care from nurses and doctors, by empowering patients towards self-management and by tracing defaulters, well-organised community support initiatives are a vital part of any sustainable public-sector ART programme.

**Conclusions:**

The review demonstrates that community support initiatives are a potentially effective strategy to address the growing shortage of health workers, and to broaden care to accommodate the needs associated with chronic HIV/AIDS. The existing evidence suggests that community support programmes, although not necessarily cheap or easy, remain a good investment to improve coverage of communities with much needed health services, such as ART. For this reason, health policy makers, managers, and providers must acknowledge and strengthen the role of community support in the fight against HIV/AIDS.

## Background

Sub-Saharan Africa remains the region most heavily affected by HIV, and accounted for 68% of all people living with the virus and for 72% of AIDS deaths in 2009 [1]. Over the past decade, important strides towards the long-term management of the HIV/AIDS epidemic in sub-Saharan Africa have been made. Currently, an estimated 3.9 million people living with HIV/AIDS (PLWHA) have started antiretroviral treatment (ART) in the region [1]. The short-term results of these ART programmes are promising. A recent review by Barth and colleagues [2] has demonstrated that the proportions of patients with on-treatment success after 2 years of first-line therapy are comparable to those from developed countries.

However, these preliminary outcomes do not warrant complacency. First, the total number of people accessing ART in sub-Saharan Africa masks wide variation in the progress made towards universal access in these countries, with Botswana and Namibia leading the path by reportedly having in excess of 85% of those in need of ART, while Mozambique, Zimbabwe, and South Africa close up the ranks with only 51–56% of those in need of access to treatment [1,3-6]. Second, a recent review by Rosen and colleagues [7] has indicated that African HIV/AIDS treatment programmes have retained only about 60% of their patients in care at the end of 2 years of treatment. However, long-term retention of patients in treatment programmes is a prerequisite for achieving any adherence at all and thus conditional to durable ART success.

Scaling up ART to achieve universal coverage and simultaneously retaining these patients in care requires strengthening or even transformation of sub-Saharan African health systems and their ART programmes. Currently, a number of programme characteristics and health system constraints severely hamper successful and sustainable treatment scale-up in the region. Five inter-related challenges are increasingly cited in health policy literature [8-11]: (1) lack of integration of ART services into the general health system; (2) the growing need for comprehensive care to address the psychosocial and economic dynamics of HIV/AIDS; (3) the need to empower patients on ART towards self-management; (4) the importance of defaulter tracing to improve retention in care; and (5) the crippling shortage in human resources for health.

The heavy HIV/AIDS burden, together with the urgency to increase patient numbers, has resulted in a strong vertical approach to programme implementation [12]. Ample research has shown that, in most countries, there has been hitherto only minimal *integration* of ART services into other district-based primary health care (PHC) services (e.g. TB care) [10,13-15]. This approach might divert scarce resources away from other vital PHC services, and subsequently create the risk of ART facilities becoming “islands of excellence in seas of under provision” [16].

ART has transformed HIV/AIDS into a manageable – though still incurable – chronic illness, which renders chronic disease care a necessity for any ART programme to be a durable and sustainable success [17,18]. In addition to the medical effects of the epidemic, it also has clear socioeconomic and psychological dimensions, therefore, any successful solution to the epidemic needs to address this multidimensionality accordingly. Consequently, he implementation of the ART programme should not only be well integrated into the PHC system, it should also be *comprehensive* in addressing the social, psychological and economic dimensions of HIV/AIDS care (e.g. social support, socioeconomic status, education), to break the cruel cycle of social and economic poverty, high-risk sexual behaviour, and further transmission of HIV/AIDS [19-21].

Closely related to the above, recent studies have indicated that, especially in resource-limited settings, PLWHA on ART should be empowered towards self-management of their chronic illness [22,23]. In practice, this *patient empowerment* entails a wide range of educating and counselling activities that are aimed at increasing HIV/AIDS and ART literacy and chronic disease management skills. However, recent studies have suggested that overburdened health staff often have difficulty in conveying the practical skills required for practicing a more patient-centred and less technical model of patient care that is aimed at empowering PLWHA for informed day-to-day decision making [22].

When comprehensive care and patient self-management fail, patients discontinue treatment and develop rapid viral rebound and loss of CD4 T lymphocytes [24], which significantly increases the risk of drug resistance and treatment failure. In addition, patients with clinical AIDS who discontinue ART will probably die within a relatively short time [24]. In sub-Saharan Africa, however, resources are scarce and the health systems overburdened, which leaves little time and resources *to trace defaulting patients*.

Against the daunting challenge to move simultaneously beyond a single-purpose, vertical programme towards an integrated PHC approach, provide comprehensive HIV/AIDS care, empower patients towards self-management and trace defaulting patients, one could well ask: *who will do the job*? [25] The inadequate supply and poor retention of skilled health professionals is deplored as the single most serious obstacle for implementing the national treatment plan in sub-Saharan Africa [26]. Thus far, the largely doctor- and nurse-driven implementation of ART has become increasingly unable to bridge the gap between the ART clinics and the vast numbers of patients in need of treatment.

Recent studies have promoted task-shifting as a key strategy for overcoming the human resources bottleneck [27,28]. In 2008, the WHO defined task shifting as “the rational redistribution of tasks among health workforce teams. Specific tasks are moved, where appropriate, from highly qualified health workers to health workers with shorter training and fewer qualifications in order to make more efficient use of the available human resources for health” [29]. Shifting tasks from medical doctors to non-physician clinicians and from these to nurses is increasingly recognized as a necessary condition for scaling up ART in sub-Saharan Africa. The WHO has identified the first as task shifting type I and the second as type II. However, in a context of human resource shortages for health – for example, in 2008, four out of every 10 professional nurse posts in South Africa were vacant [30] – task shifting from doctors to nurses will probably not be a sufficient solution to the human resource crisis. Recently, health systems research has increasingly explored the potential benefits of two other types of task shifting; namely, the shifting of tasks to lay community providers and counsellors (type III) and to the PLWHA themselves (type IV) [29].

However, an extensive literature review has demonstrated that – to date – little systematic scientific research has been performed on the contributory role of these community support and expert patient structures in HIV/AIDS treatment programmes and the health system at large in resource-limited countries [20,31]. The dearth of scientific evidence on the contributory role and function of these forms of community mobilization renders a formal evaluation and scientific review of the published results of existing community support and expert patient programmes a clear research priority. The current article therefore aims to extend the current literature by synthetically reviewing the available scientific evidence on the contribution of community mobilization to ART programmes in resource-limited settings, by focusing on both (1) the programme outcomes (coverage, adherence, virological and immunological outcomes, retention and survival) and (2) the mechanisms through which community support can overcome the five above-cited challenges to sustainably scaling-up ART in high HIV-prevalence resource-limited settings.

## Methods

To assess the potential contribution of community support structures in scaling up ART, an extensive literature review was performed. As a result of the limited number of studies that have assessed the potential contribution of community support to ART programme outcomes, we decided to expand their geographical scope to all resource-limited countries.

### Search strategy

To optimally capitalize on the relatively limited scientific information on the topic, the authors decided to apply a synthetic review design, a two-step approach which combines the strengths of a systematic review – gathering quantitative evidence on the effectiveness of community support – with additional attention for the social and behavioural mechanisms through which community support can help overcome the above-cited challenges – i.e. a realist evaluation approach [32].

Firstly, the current review aims to provide an exhaustive overview of the scientific evidence of the contribution of community support to ART programmes in resource-limited settings. To assess the achievements of community support programmes in producing favourable ART outcomes, we systematically reviewed published work (including e-publication ahead of print) for the period from November 2003 to December 2011 in accordance with the PRISMA guidelines [33]. We used the following medical subheading terms and text strings: “HIV” OR “AIDS” AND “sub-Saharan Africa” OR “sub-Sahara Africa” OR “Southern Africa” OR “resource-limited country” AND “antiretroviral therapy” OR “ART” OR “HAART” AND “community support” OR “community health worker” OR “community care giver” OR “lay health worker” OR “DOT-HAART” OR “treatment buddy” OR “adherence supporter” OR “peer health worker” OR “expert patient” to identify research articles and policy documents on the achievements of ART-related community support programmes in the ISI Web of Knowledge, Science Direct, BioMed Central, OVID Medline, PubMed, Social Services Abstracts, and Sociological Abstracts. Relevant literature was also identified by checking the websites of the WHO, the World Bank, UNAIDS, and Health Systems Trust. Government publications and institutional reports released by non-governmental organizations and academic research centres were gathered. Finally, we checked the reference lists of the above cited sources for potentially relevant books, reports and papers.

In order to merge systematic review standards with the realist evaluation approach in a second step, the latter should be applied after finishing the review in accordance with the PRISMA guidelines. In this second stage, we search for the pathways (‘why’- and ‘how’-questions) though which community support – as described in the selected studies – can impact ART programme delivery and outcomes. This requires in-depth analysis of the argumentation by the selected authors with regards to the mechanisms, contexts and outcomes of community support initiatives as part of ART programmes in resource-limited settings.

### Selection criteria

As the selected topic is innovative and the number of available scientific studies assessing the contribution of community support to ART programmes is thus rather limited, we opted to include studies applying a wide range of research designs and community support typologies.

We included all quantitative and qualitative (English-language) research papers on the selected topic, as different perspectives on a similar topic are often complementary. We included randomized controlled trials, studies utilizing a comparison group (including pre-test, post-test design), retrospective cohort studies, descriptive studies, and qualitative studies.

Secondly, with regard to the study population, we included all studies whose study population consisted of HIV/AIDS-patients enrolled in an ART programme in a resource-limited setting and not purposively selected for being on second-line treatment. ART was defined as treatment with at least three active antiretroviral medications, typically two nucleoside or nucleotide reverse transcriptase inhibitors (NTRI) plus a non-nucleoside reverse transcriptase inhibitor or a protease inhibitor or another NRTI called abacavir. We excluded articles which exclusively studied child or adolescent populations or articles reporting the accomplishments of ART programmes in resource-rich settings.

Thirdly, we opted for a broad perspective when assessing the types of community interventions included in the review. For inclusion, the studies had to comprise (1) lay members of the community (2) who enjoyed no or only limited training and (3) fulfilled a more or less organised role or function in the ART programme. This resulted in a wide range of community support initiatives (e.g. community health workers, community care workers, lay health workers, treatment buddies, field officers, peer educators/counsellors, adherence supporters, etc.). These broad criteria were chosen to capture all available evidence on the contribution of community support to ART delivery and outcomes in resource-limited settings. We did not include community-based ART programmes that lacked a clear community-based support component.

As the current review study is interested in both the impact of community support on programme outcomes and the mechanisms through which community support can overcome the five above-cited challenges to sustainably scaling-up ART in resource-limited settings, all studies that reported any type of outcome measure related to ART (including access, coverage, adherence, retention, virological and immunological outcomes, and survival), as well as all studies describing the context in which and the mechanisms through which community support can impact ART delivery and outcomes, were included.

### Study selection

**Figure 1:**
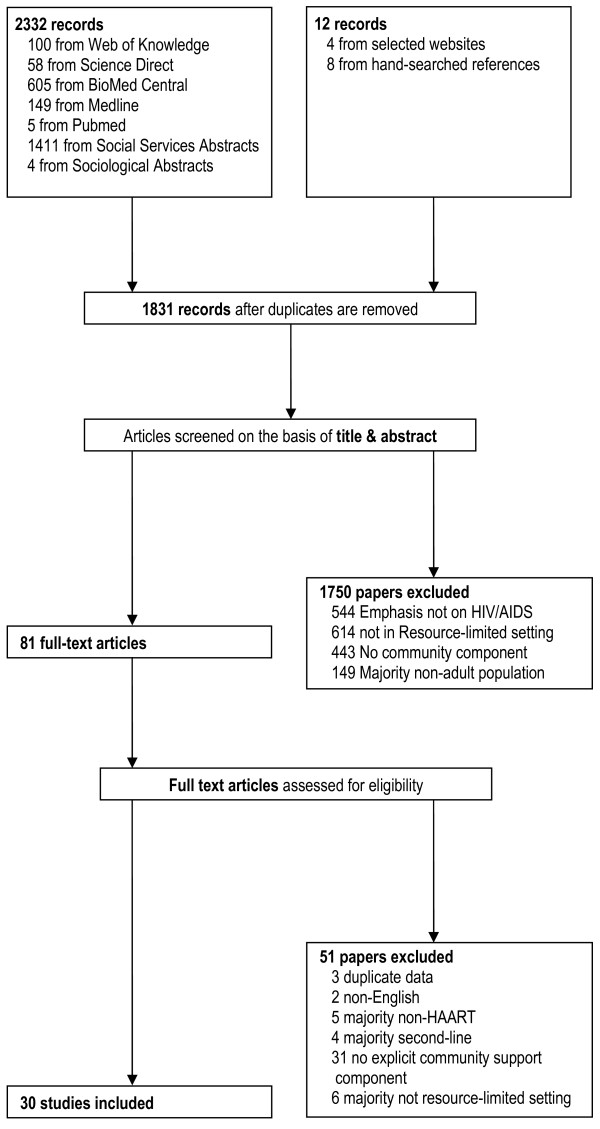
Search strategy and study selection using the PRISMA guidelines.

In accordance with the PRISMA guidelines, we first excluded all duplicates from our total of 2344 selected records. Secondly, two researchers independently reviewed all titles of the identified research papers and reports (1831 titles on community support and ART), with any differences being discussed and resolved. Of the remaining articles and reports (242 abstracts), the abstracts were assessed. In the third and final step, the authors retrieved and independently reviewed the full-length paper (81 papers) to screen it according to criteria of content (providing relevant information the impact of community support on ART outcomes (see above)) and quality (i.e. are the study design, data collection methods, sampling strategy and analytic approach apparent and appropriate; is the context described sufficiently and the range of missing data acceptable and in accordance with the methodological requirements of a systematic review as specified by Mckee & Britton, Oxman, and Walsh? [34-36] (see Figure 1)). This resulted in 29 full-text articles and one policy report, a total of 30 publications, assessing the contribution of community support initiatives to the health of HIV patients on ART in 18 different resource-limited countries (Table 1) [20,21,23,27,37-62].

**Table 1:** Summary of studies assessing the impact of community support on ART programme delivery and outcomes in resource-limited settings

**Study**	**Country**	**Research design**	**N**	**Period**	**Title of lay providers**	**Results**	**Study limitations**
Abaasa	Uganda	Retrospective cohort study	897	18 mo	Field officers	The AIDS Support Organization (TASO) ART programme displays good adherence and survival	- Retrospective design: not all potential confounders included
(2008)							
							- Self-reported adherence and the risk of social desirability bias
Assefa	Ethiopia	Descriptive study	NA	>36 mo	Health extension workers	Substantial expansion of HIV/AIDS and ART services in resource-limited context	- Descriptive study design: no causal relationships
(2009)							
							- No measurement of the extent of the impact of community support
							- Secondary and incomplete data
Bedelu	South Africa	Descriptive study	1025	20 mo	HIV/AIDS counsellors & CHWs	MSF programme using task-shifting and community support achieved near universal coverage without compromising quality of care	- Descriptive study design: no causal relationships
(2007)							
							- No measurement of the extent of the impact of community support
Benavides	Uganda	Descriptive report	5854	NC	Field officers	Field officers encourage adherence, refill medications and promote family support contributing to TASO’s ART program’s outcomes (adherence rates > 95% , reducing mortality by almost 90% )	- Descriptive study design: no causal relationships
(2006)							
							- No measurement of the extent of the impact of community support
Celletti	Brazil, Ethiopia, Malawi, Namibia, and Uganda	Desk review, observation & key informant interviews	NA	NA	CHWs	Under certain conditions, the delegation of specific tasks to CHWs can increase access to HIV services and improve quality of care.	- No clear literature search strategy
(2010)							- No clear qualitative methodology (selection of informants, data collection & analysis)
Chang	Uganda	Retrospective cohort study	360	24 mo	Peer health workers	Good adherence and survival in community- and faith-based HIV/AIDS care programme	- Retrospective study design
(2009)							
							- Reliance on clinical and programmatic records
							- Underestimation of survival because of lost-to-follow-up rates
							- Outcome measurements not measured at exact time intervals
Chang	Uganda	Cluster-randomized trial	1336	>22 mo	Peer health workers	A peer health worker intervention was associated with decreased virologic failure, but did not affect cumulative risk of virologic failure, adherence measures or short-term virologic outcomes	- Limited generalisability: mobile clinic setting
(2010)							
							- Weakness of design: imbalances between clusters
							- Limited statistical power
Cohen	Lesotho	Descriptive study	5376	40 mo	HIV/AIDS counsellors	Lay counsellor-supported testing and counselling, adherence and case management produced favourable outcomes	- Descriptive study design: no causal relationships
(2009)							
							- No measurement of the extent of the impact of community support
Etienne	Kenya, Rwanda, Uganda, Tanzania, Zambia, Nigeria, Haiti, and Guyana	Descriptive study	13391	12 mo	Adherence supporters	Adherence counselling, structured treatment preparation, community home visits, and supportive supervision by community nurse significantly reduced the loss to follow up.	- Descriptive study design: no causal relationships
(2010)							
							- Potential selection bias at the facility level
Gusdal	Ethiopia & Uganda	Qualitative study	118	NA	(peer) HIV/AIDS counsellors	Peer counsellors served as facilitators of adherence, role models and bridges to the health system	- Selection bias: no information on patients lost-to-follow-up
(2011)							- Saturation of data not achieved
Hermann	Ethiopia, Malawi, and Uganda	Desk review & descriptive field research	NA	NA	CHWs	Present CHW programmes are essential for ART scale-up and comprehensive care but have insufficient attention to quality supervision, continuous training,and the life experience of PLWHA	- No clear literature search strategy
(2009)							
							- No clear methodology for the literature analysis
Idoko	Nigeria	Quasi-experiment	175	12 mo	DOT ART supporters	Patients accessing treatment support (daily/ twice weekly/weekly observed therapy) demonstrated better treatment outcomes compared to control group	- Limited genralisability: one facility
(2007)							
							- Small sample size: limited statistical power
							- No statistically significant differences
Igumbor	South Africa	Retrospective patient record review	540	9 mo	adherence supporters	Patients with community adherence support maintained a suppressed VL and remainedin care for a longer period as opposed to patients lacking this support	- Retrospective study design (no causal relationships)
(2011)							
							- No measurement of the extent of the impact of community support
Jaffar	Uganda	Cluster-randomised equivalence trial	1453	42 mo	Field officers	Home-based HIV care was as effective as facility-based care: similar virological failure and mortality rates	- Refusals and withdrawals can create selection bias
(2009)							
							- Weakness of design: imbalances between clusters
Kabore	Lesotho, South Africa, Namibia, and Botswana	Observational cohort study	377	18 mo	CHWs, HBC volunteers & adherence supporters	Community support was associated with more rapid and greater CD4 increase and higher levels of adherence. Home-based care and/or food support was associated with greater improvements in HRQoL.	- Observational study design: patients select to receive support
(2010)							
							- Potential selection bias
							- High rate of patients who were lost-to-follow-up
Koenig	Haiti	Descriptive study	1050	12 mo	CHWs	DOT-HAART using CHWs resulted in good viral suppression and high survival rates	- Descriptive study design: no causal relationships
(2004)							
							- No measurement of the extent of the impact of community support
Kunutsor	Uganda	Randomized controlled trial	174	7 mo	Adherence supporter	Patients with an adherence supporter had over 4 times the odds of achieving optimal adherence and were more l ikely to be on time for their clinical appointments	- Limited genralisability: one facility
(2011)							
							- Potential selection bias (of highly motivated patients)
							- Limited statistical power (adherence measurement method)
Morris	Zambia	Descriptive study	NA	36 mo	Peer health workers	Improved clinical care quality despite growing patient volumes	- Descriptive study design: no causal relationships
(2009)							
							- No measurement of the extent of the impact of community support
							- Programme’s intensive use of resources
Mukherjee	Haiti	Descriptive study	1500	12 mo	CHWs	Home-based adherence support from a network of CHWs produces low rates of treatment failure	- Descriptive study design: no causal relationships
(2006)							
							- No measurement of the extent of the impact of community support
Mukherjee	Haiti	Descriptive study	NA	NA	CHWs	CHWs facilitate the uptake of PHC services, including by the most vulnerable households	- Descriptive study design: no causal relationships
(2007)							
							- No measurement of the extent of the impact of community support
							- Limited representativeness of qualitative results
Muñoz	Peru	Quasi-experiment	120	12 mo	CHWs & DOT ART supporters	CASA (community-based accompaniment with supervised antiretroviral) participants reported better clinical and psychosocial outcomes compared to control group	- Small sample size
(2010)							
							- Potential selection bias (confounding differences)
							- Limited generalisability
Nachega	South Africa	Randomized controlled trial	274	24 mo	DOT ART supporter	DOT-ART was associated with greater median CD4-cell count and better survival rates, but not with improved virological outcomes	- Limited genralisability
(2010)							
							- Limited time frame of the intervention
							- Relatively low incidence of AIDS-defining illness and death
Pearson	Mozambique	Randomized controlled trial	350	12 mo	Peer DOT ART supporters	Intervention participants demonstrated significantly higher ART adherence	- Initial phase of ART programme (highly motivated patients)
(2007)							
							- No blinding of the participants and the study personnel
							- Self-reported adherence measure
							- Limited generalisability: one facility
Rich et al.	Rwanda	Retrospective medical record review	1041	24 mo	CHWs	Community based ART produced very high levels of retention and large increases in CD4 cell count. However, the relative impact of the different components of the program could not be determined.	- Descriptive study design: no causal relationships
(2012)							
							- No measurement of the extent of the impact of community support
							- Low data completeness for key variables
							- Potential selection bias
Selke	Kenya	Randomized controlled trial	208	12 mo	CCCs	Community-based care by PLWAs resulted in similar clinical outcomes as standard care but with half the number of clinical visits	- Limited generalisability
(2010)							
							- Selection bias: different sublocations & only adherent patients included
							- Small sample size (limited statistical power)
Weidle	Uganda	Nested randomised trial	987	12 mo	HIV/AIDS counsellors & Field officers	Group education, personal adherence plans, a medicine companion and home-delivery of ARVs by lay counsellors achieved good ART adherence and reponse	- Selection bias: participants selected from a community AIDS organisation
(2006)							
							- Limited statistical power
							- No measurement of the extent of the impact of community support
Wools-Kaloustian	Kenya	Quasi experiment	NA	24 mo	CCCs	An ART delivery model that shifts patient monitoring and ARV dispensing to CCCs is both acceptable and feasible	- Limited generalisability: one facility
							- Programme’s intensive use of resources (PDAs & training)
(2009)							
Wouters	South Africa	Retrospective cohort study	371	24 mo	CHWs & adherence supporters	Community support predicted better viral suppression and immunological restoration	- Study design: patients select to receive support
(2009)							
							- Potential selection bias (confounding differences)
							- Underestimation of survival because of lost-to-follow-up rates
Wouters	South Africa	Retrospective cohort study	371	24 mo	CHWs & adherence supporters	Community support initiatives (CHWs and support groups) positively impacted disclosure to family members	- Study design: patients select to receive support
(2009)							
							- Potential selection bias (confounding differences)
Zachariah	Malawi	Quasi-experiment	1634	20 mo	HBC volunteers.	Community support was associated with significantly lower death rate and better ART outcomes	- Study design: not possible to know the exact reasons for the observed differences
(2007)							

NA = not applicable, NC = not clear, mo = months.

### Analysis

In accordance with the methodological literature [63], we performed a systematic content analysis to produce a concise summary (Table 1) of the overall effect of community support interventions on ART programmes outcomes. However, Van de Knaap et al. [32] and Forbes & Griffiths [63] have already indicated that such a systematic review only demonstrates whether a policy intervention works or not, and not why or how it works. Therefore, the current review also used a realist evaluation strategy to assess the context of the studies and the mechanisms through which these community support initiatives can address the above-cited ART programme challenges, and thus contribute to a sustainable ART scale-up in resource-limited countries [32,63,64].

## Results

### Description of the community support programs

The selected articles reported on the outcomes of 22 different programmes in 18 countries. Only eight of the 30 selected papers (27%) were published in 2007 or earlier. All other papers were published more recently, underlining the increasing relevance of and attention for this topic in health systems research.

As a consequence of the broad selection criteria – aiming to assess the impact of community support on ART programme outcomes as well as obtain a comprehensive overview of the potential contribution of these community support initiatives in addressing the five cited challenges to a sustainable ART scale-up – the 30 studies used a wide range of methodological designs: nine descriptive studies, five (cluster/nested-)randomised controlled trials, four quasi-experiments, five retrospective/observational cohort studies, and two qualitative studies. By offering relevant information on the sample size, research design, time frame and study limitations, Table 1 provides insight into the scientific and methodological quality of the included studies.

Table 1 further demonstrates that the selected studies represent a wide range of community support providers that are involved in ART-related activities. In addition, different studies often use alternative names for the same type of community support provider. Our review identified the following nine types. The first type of community support providers are *community health workers* (CHWs) (11 studies). CHWs can be defined as “non-professional cadres of health workers who undertake short course training and work within their own communities to complement and support the services provided by other health workers” [43,65]. This definition also includes ‘accompagnateurs’, as developed by Partners In Health (PIH) [40,50]. Secondly, two studies employed *community care coordinators*, which can be described as PLWHA who are trained to perform the tasks of CHWs [60,61]. Very similarly, three studies assessed the contribution of *peer health workers*, defined as CHWs who are HIV positive and whose role it is to conduct adherence counselling and provide health education and psychosocial support [27,44,45]. Fourthly, a number of studies employed *field officers* as a means for community mobilisation: field officers can be defined as trained lay workers who support drug delivery and monitor patients. This is very similar to the definition of CHWs, but field officers generally enjoyed formal education in social sciences or education, enabling them to embrace a more holistic approach with sufficient attention for the psychosocial aspects of health and illness [38,41,48]. The fifth type of community support initiative, *health extension workers*, are recruited from the community and trained to manage operations of health posts, conduct home visits and outreach services to promote preventive health actions, refer cases to health centres and follow up on referrals [37,66]. Four studies employed (peer) *HIV/AIDS lay counsellors*, whose tasks included managing HIV testing, providing pre-ART training and ART adherence support, searching for patients eligible for ART and tracking defaulting ART patients [39,42,54,55]. The seventh type of community support strategy is *Directly Observed Therapy (DOT) for ART*, employing a community member close to the patient to directly monitor the daily medication intake – a strategy modelled after an effective method for controlling tuberculosis [47,67]. The eighth group of community support providers, labelled *adherence supporters*, are trained to promote healthy behaviours, provide HIV/AIDS&ART-related knowledge and skills and promote adherence by providing psychosocial support [20,21,46,49,56,57]. The final form of community support is labelled *home-based care volunteers*. These community members are trained to provide ART adherence counselling and perform a wide range of home-based care activities [62].

The wide range of tasks performed by community members, combined with the different descriptions for similar activities indicate that there is a need for further conceptual work in order to clearly establish a typology of these community support initiatives applicable in the field as well as in research studies on the topic. The current review study aims to explore the common ground between all these different types of community support in order to provide a comprehensive overview of the potential contribution of these different community support initiatives to successfully scaling-up ART in developing settings. However, it is vital to recognize – and take into account – this diversity in community support initiatives and its descriptions (displayed in Table 1) when interpreting their contribution to ART programme delivery and outcomes in high HIV-prevalence resource-limited settings.

The literature search indicated that there is not much scientific evidence of the explicit use of PLWHA in ART programmes. We found only 12 articles that reported the use of HIV-positive individuals in ART care [23,27,39,42,44-46,55,58,60-62], of which, only seven specifically targeted PLWHA as providers of community support in ART programmes [42,44,45,55,58,60,61]. Despite this wide range of community support providers that are involved in ART-related activities, it is useful to search for common roles and functions in scaling up ART in resource-limited settings to obtain an evidence-based overview of their relative contribution.

### Impact on programme outcomes

A review of the literature (Table 1) on the association between community support and ART programme outcomes clearly indicates that the community can have a contributory role in scaling up ART in resource-limited settings [20,23,41,42,45,47,48,50,52,53,62,68]. Almost all selected studies reported an unambiguous positive impact of community support on a wide range of aspects of the ART programme. First of all, a number of studies indicated that community support can aid in expanding the *access to* and increasing the *coverage of ART programmes* in resource-limited settings [23,27,37,38,40,42,43,51,55]. In addition, the services provided by these community support initiatives have been shown to increase *adherence levels*[41,49-51,54-58]. Thirdly, a number of studies demonstrated that the community component significantly improved the *virological and immunological outcomes* of the programme: patients with this kind of support achieved higher rates of virological suppression and immune restoration than patients lacking this support [20,21,23,27,38-40,43-45,47-52,54,56,59-62]. Finally, the results displayed in Table 1 also indicate that community support initiatives can improve *levels of patient retention*[39,42,46,48,56,59,61,62] and increase *rates of survival* in ART patients in high HIV-prevalence, resource-limited settings. [39,41,48,53,59,62]. Unfortunately and although the general trend is evident, it was not possible to quantitatively estimate the mean effect of community support initiatives on the selected ART programme outcomes because of the fact that the selected studies were limited in number and displayed a wide range of programme and research designs.

### Contributory role

The current review aimed to collect the scientific evidence for the potential of community support initiatives to address the above-cited barriers to sustainable ART scale-up in a resource-limited setting, namely: (1) the lack of integration of ART services into the general health system; (2) the growing need for comprehensive care addressing the psychosocial and economic dynamics of HIV/AIDS; (3) the need to empower patients on ART towards self-management ; (4) the importance of defaulter tracing to improve retention in care; and (5) the crippling shortage in human resources for health in contrast to the growing caseload of people to be maintained on ART in the long term. To combat the HIV/AIDS epidemic effectively in high-HIV-prevalence, resource-limited countries, we thus need more care (i.e. integrated and comprehensive chronic disease care, patient empowerment and defaulter tracing) for more people (i.e. universal access) in a context of limited financial and human resources. *How* and *why* community care providers – according to the selected studies – can contribute to sustainably and successfully scaling up ART in resource-limited settings, while maintaining a high quality of care, is demonstrated below.

#### Bridging the gap

Out of a total of 30 studies that assessed the impact of community support initiatives on ART programme outcomes, 18 (60%) specified the ability of community care providers to integrate HIV/AIDS care into the general PHC system as a potential contribution of these lay providers to a sustainable ART-scale-up [20,21,23,37-40,42,44,46,49-52,55,58,59,62]. A study by Koenig and colleagues [50] showed that community support initiatives – such as CHWs, adherence supporters and community care workers – can act as multipurpose health workers who could link HIV/AIDS care to other equally important PHC programmes (e.g. TB control). In this way, community support initiatives can – as shown by Zachariah et al. [62,69], Bedelu et al. [42] and Gusdal et al. [55] – bridge the gap between the patient and various health care programs (TB, HIV/AIDS, malaria), without compromising the quality of care. In this regard, Muñoz and colleagues [52] have demonstrated that community health workers are vital for the most vulnerable patient groups as these lay providers can serve as a liaison with formal health services. The evidence suggests that, by acting as multipurpose health workers, community care providers can help integrate the ART programme into the general health care system, thus preventing the programme from becoming a simple add-on to the health system, and ensuring that the massive additional investment in the health system brought about by the ART programme can aid in strengthening the health system as a whole [70]. By mobilisation of the community in the fight against HIV/AIDS, public health systems can potentially overcome the first barrier to successful ART scale-up and bridge the gap between the “islands of excellence and the seas of under-provision”[16].

#### Providing psychosocial care

Almost all articles (29 or 97%) on the potential contribution of community support to ART scale-up stressed the capacity of community care providers to broaden HIV/AIDS care beyond mere medical care tasks, by providing social support and counselling [20,21,23,27,37-57,59-62]. In this way, community support initiatives meet the emerging needs associated with chronic AIDS care, where, as a result of the growing shortages of health professionals, their roles are becoming progressively limited to technical medical and nursing tasks. The empirical evidence of Table 1 demonstrates that the psychosocial adherence support provided by community support providers can have a positive impact on ART programme outcomes. Wouters and colleagues [20,21,71,72] have demonstrated that the social and motivational support of community initiatives, such as treatment buddies, CHWs and patient support groups can stimulate disclosure to the public and family members, and positively influence clinical ART outcomes. The continuous adherence support of treatment buddies, the motivational support of CHWs, and the sharing of experiences in patient support groups independently leads to better viral suppression and immunological restoration throughout the first 2 years of treatment [20,72]. In addition, a retrospective study by Etienne and colleagues [46] has shown that a range of community support activities significantly improved patient retention rates in eight resource-limited countries. These findings were supported by a Ugandan study by Chang et al. [45] that provided empirical evidence for the capacity of community support initiatives to combat treatment fatigue and thus promote the sustainability of ART in low-resource settings. This evidence suggests that community support initiatives can be a valuable component in offering comprehensive HIV/AIDS care, that is, with sufficient attention to the multifaceted nature of the illness and its treatment.

#### Empowered ART patients

Although a comprehensive approach to ART acknowledges the multidimensional nature of HIV/AIDS and the associated care needs, one can also focus on an individual’s strengths, resources and ability to address the challenges of HIV/AIDS and ART. A number of studies (19 or 63%) have used such a patient-centred chronic disease care model of care to explore the potential contribution of community support providers on patients’ ability to manage their own illness [20,23,27,37-40,42,43,47,49,52,54,55,58,59,61,62,67]. Bedelu et al. [42], Celetti et al. [43], Bendavides & Caffrey [38] and Cohen et al. [39] have reported that CHWs can help patients to develop the self-management skills that are needed to take well-informed decisions regarding their health and treatment, as well as articulate their needs and negotiate with health providers in the public sector about their rights and the quality of treatment they receive. In practice, Kabore and colleagues [49] have revealed that the standard package of community-based services aimed at empowering patients towards self-management includes home-based care, nutritional advice, drug literacy training, prevention education, management of ART side-effects, and general treatment guidance.

In addition, HIV/AIDS is a chronic condition that has resulted in a growing pool of people living with the disease. The concept of using the personal experiences of these expert patients has the potential to be one important building block for chronic care programmes in resource-limited settings [23,61]. Despite the fact that ART programmes in industrialised countries consider the life experience of PLWHA to be an important asset in HIV/AIDS care [23], relatively few studies (12 or 40%) have explored their potential contribution in resource-limited settings. The current body of research on the contribution of expert patients as peer educators and counsellors nevertheless indicates that these community support providers are a valuable resource to educate and support HIV/AIDS patients to become competent managers of their own health and treatment [23,27,39,42,44-46,55,58,60-62].

#### Defaulter tracing

Rosen and colleagues [7] have identified loss-to-follow-up as the most important cause of patient attrition. Patients who default usually develop rapid viral rebound and often die within a short time period, therefore, it is vital to minimise patient attrition by preventive and reactive measures. A series of recent studies has demonstrated that psychosocial support and regular home visits by CHWs and peer adherence counsellors have acted as powerful preventive actions against patient attrition [23,40,49,54,56,59,61]. When preventive measures fail and patients are not presenting themselves at the facility, it is vital to bridge the gap rapidly between the facility and the community, and actively track these defaulters. Several studies have indicated tracing defaulters as one of the standard tasks of community support providers [37,39,42]. When looking at the results, Kabore and colleagues [49] have reported that defaulter tracing by CHWs is associated with better ART outcomes in rural Malawi. Out of a total of 29 studies that assessed the contribution of community support in ART outcomes, 17 (or 579%) indicated community support providers’ ability to reach out into the community and prevent loss-to-follow-up or track defaulting patients [20,23,37,39,40,42,43,45,46,48,49,52,54,56,58,61,62].\

#### Community as a resource

The evidence displayed in the literature suggests that community mobilisation can at least be part of the solution to the cited human resource shortages. A total of 21 studies (70% of selected studies) demonstrated the feasibility and effectivity of an ART programme with a clear CHW component [20,21,23,27,37-39,41-48,54,55,57,60-62]. A multi-country study by Celetti et al. [43] has demonstrated that the deployment of CHWs represents an effective and sustainable response to the health workforce shortages in many sub-Saharan countries. Jaffar and colleagues [48] have demonstrated that HIV/AIDS care with lay health workers supporting drug delivery is as effective as a nurse-led and doctor-led clinic-based strategy in terms of virological suppression and patient survival. These findings suggest that, in regions where nursing staff as well as doctors are in short supply, an HIV-care strategy using lay workers can enable improved and equitable access to HIV treatment [48].

In addition to this potential expansion of the health workforce, recent studies by Wouters et al. [20] and Zacharia et al. [62] have stressed the importance of transforming social structures to create an environment in which individuals cannot only protect themselves against HIV infection, but also count on the support of their fellow community members when they would need care – so-called AIDS-competent communities. The creation of AIDS-competent communities can potentially be a good investment to use optimally the limited funding available to developing countries to address the epidemic. In addition, the psychosocial care provided by community support initiatives such as ART support groups and treatment buddies can potentially increase the effectiveness and efficiency of public-sector treatment efforts.

These studies suggest that, by providing comprehensive care to more patients and tracing defaulters, community support initiatives can improve coverage and quality of care simultaneously [20,21,23,27,37-39,41-46,48,54,61,62]. The scientific evidence thus consistently demonstrates that communities can be considered an under-exploited resource in building and strengthening the chronic disease care capacity needed to scale up ART successfully in the public sector, as well as to integrate a comprehensive HIV/AIDS strategy into the general health system.

### Not a panacea

The vast majority of studies have indicated that community support providers have the potential to become essential players in the provision of HIV/AIDS care in particular and form part of a broader mobilisation of community and non-governmental participation in the health system at large [10]. However, the literature review did not only produce positive outcomes. Ford and colleagues [73] systematically assessed a series of randomised control trials that tested the outcomes of DOT-ART. Over half of the studies were performed in the United States, but five included data from resource-limited settings. The article showed no statistically significant benefit of DOT-ART over self-administered treatment, which questions the concurrent scientific results that indicated strong positive community support outcomes. However, the results of Ford’s review should not be viewed as evidence against community support, but rather as evidence that a particular paternalistic and disempowering approach to community support does not necessarily produce positive treatment outcomes. To capitalise optimally on the potential contribution of the community to a sustainable ART programme, community support should not render the patients incapable and thus in need of care, but rather support patients to take up responsibility. Our findings indicate that community support can be a crucial part of such a health-enabling community that is designed to empower the patients to self-manage their illness actively and durably.

A number of studies raised concerns about the quality and sustainability of the community support programmes [23,39,55,61]. Most of the articles in our review focused their attention on showing that the use of lay health workers does not compromise the quality of HIV/AIDS care. The vast majority of reviewed studies concluded that this fear did not materialise during the observed time period, but these favourable outcomes do not warrant complacency [27,44,45,61]. Hermann and colleagues [23] have reported that community support programmes often devote insufficient attention to CHW retention, quality supervision and continuous training, which jeopardises the quality of these programmes over time. Several programmes have indicated that CHWs cannot be retained in the long term if they do not receive adequate remuneration [55]. A more formal recognition of these initiatives as an integral part of the health systems is therefore urgently required. The real cost of community support programmes also includes the qualified human resources needed to supervise and train these new community health worker cadres [23]. The above indicates that community mobilisation is probably a necessary component of scaling up ART in sub-Saharan Africa, but not necessarily a cheap or an easy one.

To capitalise fully on the communities’ ability to play a contributory role in durably scaling up ART, a supportive policy environment and an enabling regulatory framework are needed to drive the implementation of task-shifting policies and to resource, guide and support these lay CHWs [55,74]. Scott & Shanker (2010) reported in their article on an Indian CHW programme that the programme under study was institutionally limited by (1) the outcome-based remuneration structure; (2) inadequate institutional support; (3) the rigid hierarchical structure of the existing health system; and (4) a dearth of participation at the community level [75]. Hermann and colleagues [23] have mentioned five necessary conditions for all CHW programmes to be successful, namely: (1) careful selection based on motivation and belonging to the community; (2) initial training; (3) simple guidelines and standardised protocols; (4) good supervision, support and relationships with formal health services; and (5) adequate remuneration and career structure. An additional three conditions were identified to scale up these CHW initiatives successfully in resource-limited settings. Without (1) sufficient political support, (2) a broader strengthening of the health system, and (3) sufficient flexibility and dynamism of these community initiatives, it is highly unlikely that the above-cited positive impact on ART programme outcomes can be achieved in the long term [23]. The renewed attention to community mobilisation in high-HIV-prevalence, resource-limited settings is very welcome, but researchers and policy makers should continuously devote sufficient attention to creating an enabling context for these initiatives as well as guarding the necessary quality criteria.

## Discussion

The current study gathered and assessed the available scientific evidence of the contribution of community support on ART programmes in resource-limited setting by looking at both (1) the programme outcomes and (2) the mechanisms through which community support can overcome the five often-cited challenges to sustainably scaling-up ART in high HIV-prevalence resource-limited settings – namely (1) a lack of integration between ART and other health care programmes, (2) the need for comprehensive care, (3) patient empowerment and (4) defaulter tracing, and (5) rising patient numbers combined with human and monetary resource shortages.

Although the differing research designs did not allow us to statistically compile the available evidence, the answer to our first research question appears to be unequivocally positive. Our synthetic review demonstrates that community support initiatives can positively impact ART programme outcomes in resource-limited settings. The reviewed literature reported an unambiguous positive impact of community support on a wide range of aspects, including access and coverage, adherence, virological and immunological outcomes, patient retention and survival [20,21,23,27,37-62]. The evidence suggests that a durable programme of universal access to ART will not only require a new level of performance of the regular health system, but also the mobilisation of additional human resources, namely of the community as a whole and community care givers in particular [70,76].

In addition, the review demonstrates that community support initiatives are an effective strategy to address, through purposive and well-monitored task-shifting, the chronic needs associated with the current HIV/AIDS epidemic. Given the pressure on health systems and their professional staff, the existing evidence suggests that CHW programmes, although not necessarily cheap or easy, remain a good investment to improve coverage of communities with much needed health services, such as ART [20,77]. In addition, community support initiatives can meet, within a context of scarce resources and in an integrated manner, the new psychosocial needs associated with HIV/AIDS as a life-long chronic illness, thus also improving the quality of care [20,21,62,69,72,78]. After the initial transition to life on ART, with its associated comprehensive psychosocial and economic support needs, the available evidence suggests that community support providers can guide the gradual movement into a maintenance phase of empowered living by assisting PLWHA in building the necessary motivation, support, and knowledge to sustain excellent adherence. When the comprehensive care provided by CHWs and the self-management skills developed by the PLWHA do not suffice, CHW can potentially be used to bridge the gap between the facility and the community – as recently suggested by Harries et al. (2010) – by tracing patients who would otherwise fail treatment, possibly infect others, and would most likely not survive [79]. In this way, the current synthetic review study empirically confirms the claims made by Campbell & Cornish (2010) – in their recent editorial of a supplementary issue of this journal – about the value of community mobilisation in AIDS management [80].

The available empirical evidence thus confirms the community’s ability to address five often-cited impediments to a sustainable ART scale-up [20,62,69,72]. For this reason, health policy makers, managers, and providers have to acknowledge and strengthen the role of community support in the fight against HIV/AIDS. However, community support cannot be considered as a panacea for weak health systems or a way to reduce public health expenditure. One should be aware that many of the activities carried out by community support providers should ideally fall under the responsibility of the public service. Scientific evidence of the potential of the community to assist in scaling up ART should thus not be used as an excuse for not addressing the structural deficiencies in the health sector. In addition, communities should not be considered merely as an entity to which tasks can be delegated with a view to save costs. Rather, community support initiatives – such as treatment buddies, CHWs, and expert patients – should be given a recognised role in achieving durable ART success, while the roles of health care professionals are restricted to technical medical tasks. Only in this way can community support meaningfully contribute to a comprehensive ART programme that is successfully integrated in the general district-based PHC system, and can thus achieve universal ART coverage.

The strengths of the present review include its systematic search and selection strategy and its focus on an understudied but vital component of the health system in resource-limited settings, namely the community. To the best of our knowledge, this is one of the first studies to assess, in a systematic manner, the impact of community support on ART outcomes in high-HIV-prevalence, resource-limited settings. In this way, the current review extends on the findings of the Cochrane Collaboration paper by Rueda et al., which produced similar findings based on evidence gathered in Western, developed settings [31]. However, two main limitations could affect the interpretation of this review. First, the selected studies were rather heterogeneous in design, and systematic review methodology is most successful when the selected studies used a similar research design – preferably randomised controlled trials. One must note, however, that the review findings illustrate that randomised controlled trials might not be the appropriate method for comprehensively studying community involvement in HIV/AIDS programmes. Community support providers are usually just one part of complex ART delivery models that cannot be fully captured using one particular research design. Second, as noted above, the review included a wide range of community support initiatives reducing the comparability of the outcomes. The different descriptions for often similar activities indicate that there is a need for further conceptual work in order to clearly establish a typology of these community support initiatives applicable in the field as well as in research studies on the topic. Third, it is to be expected that community support programmes that fail to have a positive impact, or even negative influence on ART programme outcomes are less likely to be reported in the scientific literature, which potentially results in a publication bias and an overestimation of the beneficial impact of community involvement.

The current review demonstrates that further large-scale and longitudinal research studies are needed to disentangle fully the complex interrelationships between various forms of community support and the multidimensional aspects of HIV/AIDS care and treatment in a variety of research settings. In as much as the preliminary study findings have described a number of encouraging aspects of community involvement, and the potential to enhance community involvement in health-related activities, further empirical research is vital to understand which tasks performed by these community support initiatives, and peer support activities in particular, contribute to achieving durable ART success. However, empirical research on the capability, efficacy and efficiency of community care providers in taking on additional roles is also needed to understand the limits to which lay workers can assume multiple roles and still perform adequately, the level of training and the structural framework that are required to fulfill these new roles and the optimal balance between general and specialist roles [10]. Further evaluation of the roles of these initiatives in HIV/AIDS care should therefore be an important research priority and should include studies in various settings and that employ alternative research designs.

## Conclusion

The current synthetic review demonstrates that community support can positively impact ART programme delivery and outcomes in resource-limited settings. The review shows that community support initiatives are a potentially effective strategy to address the growing shortage of health workers, and to broaden care to accommodate the needs associated with chronic HIV/AIDS. The existing evidence suggests that community support programmes, although not necessarily cheap or easy, remain a good investment to improve coverage of communities with much needed health services, such as ART.

However, the evidence also demonstrates that further large-scale and longitudinal research studies are needed to disentangle fully the complex interrelationships between various forms of community support and the multidimensional aspects of HIV/AIDS care and treatment in a variety of research settings. In as much as the preliminary study findings have described a number of encouraging aspects of community involvement, and the potential to enhance community involvement in health-related activities, further empirical research is vital to understand which tasks performed by these community support initiatives, and peer support activities in particular, contribute to achieving durable ART success. However, empirical research on the capability, efficacy and efficiency of community care providers in taking on additional roles is also needed to understand the limits to which lay workers can assume multiple roles and still perform adequately, the level of training and the structural framework that are required to fulfill these new roles and the optimal balance between general and specialist roles [10]. Further evaluation of the roles of these initiatives in HIV/AIDS care should therefore be an important research priority and should include studies in various settings and that employ alternative research designs.

## Competing interests

The authors declare that they have no competing interests.

## Authors’ contributions

EW initiated the paper. EW and HM undertook the literature searches and extracted data. EW wrote the draft manuscript. HM, WVD, CM and HVR provided suggestions, helped to identify relevant studies, and contributed fully to the revisions of the paper. All authors approved the final version.

## Pre-publication history

The pre-publication history for this paper can be accessed here:

http://www.biomedcentral.com/1472-6963/12/194/prepub
